# Quadruplex-duplex junction in LTR-III: A molecular insight into the complexes with BMH-21, namitecan and doxorubicin

**DOI:** 10.1371/journal.pone.0306239

**Published:** 2024-07-24

**Authors:** Stefania Mazzini, Gigliola Borgonovo, Salvatore Princiotto, Roberto Artali, Loana Musso, Anna Aviñó, Ramon Eritja, Raimundo Gargallo, Sabrina Dallavalle

**Affiliations:** 1 Department of Food, Environmental and Nutritional Sciences (DEFENS), University of Milan, Milan, Italy; 2 Scientia Advice di Roberto Artali, Cesano Maderno (MB), Italy; 3 Institute for Advanced Chemistry of Catalonia (IQAC), CSIC, Networking Center on Bioengineering, Biomaterials and Nanomedicine (CIBER-BBN), ISCIII, Barcelona, Spain; 4 Department of Chemical Engineering and Analytical Chemistry, University of Barcelona, Barcelona, Spain; 5 National Institute of Fundamental Studies, Kandy, Sri Lanka; National Cancer Institute at Frederick, UNITED STATES

## Abstract

Quadruplex-Duplex (Q–D) junctions are unique structural motifs garnering increasing interest as drug targets, due to their frequent occurrence in genomic sequences. The viral HIV LTR-III sequence was chosen as a Q–D junction model to study the affinity of the selected compounds BMH-21, namitecan (ST-1968), and doxorubicin (DOXO), all containing a planar polycyclic aromatic moiety, linked to either one short aminoalkyl or an aminoglycosyl group. A multidisciplinary approach that combines NMR spectroscopy, molecular modelling, circular dichroism (CD) and fluorescence spectroscopy was employed. The studied ligands induced moderate but clear stabilization to the Q–D junction by interacting with the interfacial tetrad. DOXO was found to be the best Q–D junction binder. Interestingly, the removal of the aminoglycosyl group significantly changed the pattern of the interactions, indicating that highly polar substituents have a stronger affinity with the exposed regions of the Q–D junction, particularly at the level of the interfacial tetrad.

## Introduction

**Fig 1:**
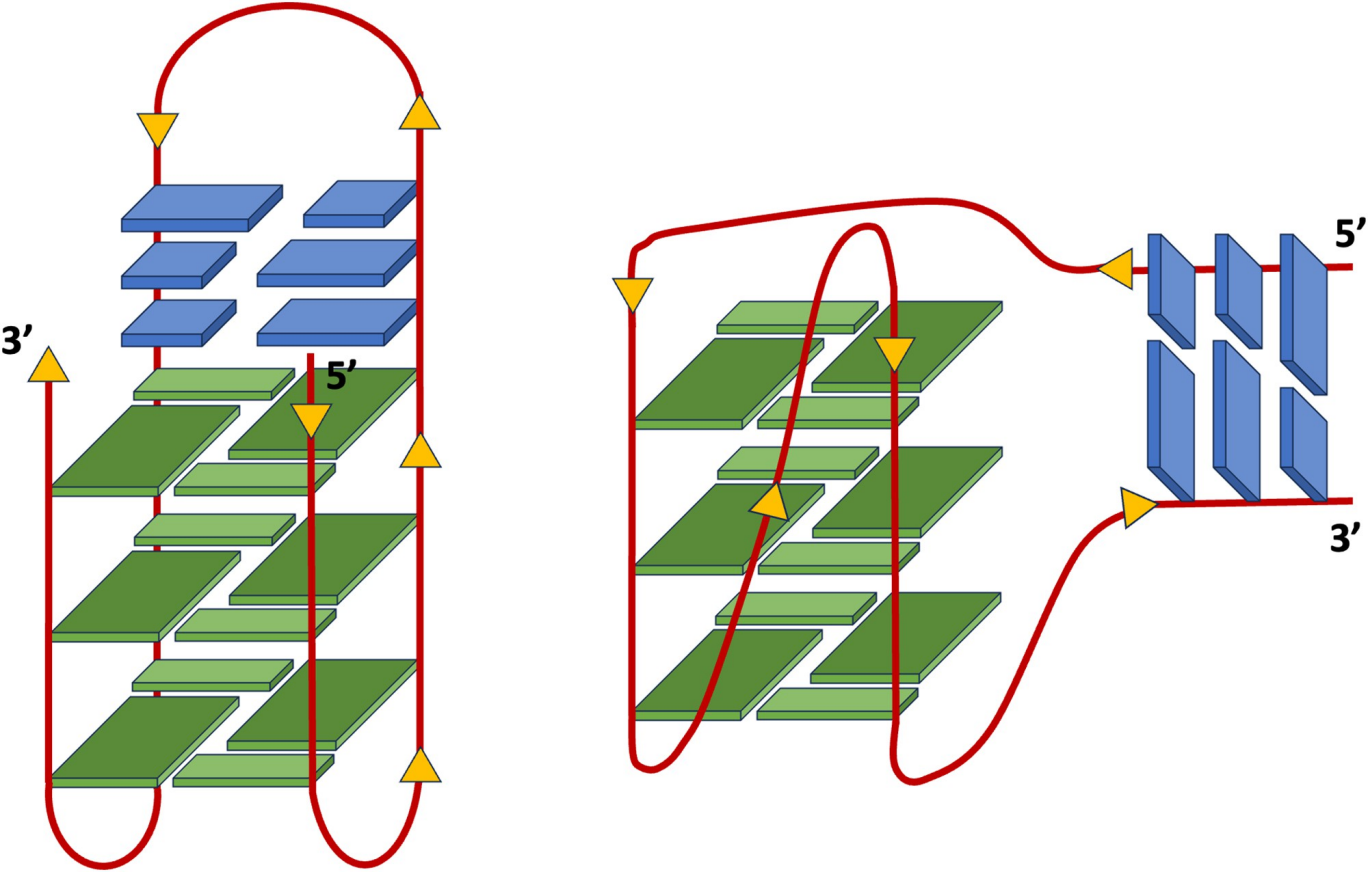
Schematic representation of coaxial (left) and orthogonal (right) orientations present in typical Q–D junctions. G-quadruplex sequences are reported in green, duplex sequences are in blue.

Sequences with four runs of G-nucleotides can fold into four-stranded quadruplex structures (G4s). The resulting stacked G-quartets are further stabilized by the coordination with monovalent cations, such as Na^+^ or K^+^, within the central channel of the G-core [1]. G4 forming sequences exert important biological roles in the regulation of various physiological processes. Due to the abundance of G-rich sequences at critical locations within the genome, such as promoter regions of human oncogenes c-Myc, c-Kit, and KRAS [1–4], these alternative nucleic acid structures have been recognized as promising therapeutic targets for small molecules. In this framework, several compounds, mostly containing flat and polycyclic aromatic systems, have been synthesized and tested as G4 binders. With their structures these ligands are able to stack the outer G-tetrads, without establishing additional interactions throughout overhang residues [5–9]. As such, selectivity towards different G4s is particularly difficult to achieve. Several approaches have been proposed to tackle this problem [10–16]. Among others, targeting the interface between quadruplexes and adjacent double-stranded regions is recently emerging as a promising strategy. As DNA is mainly found in the cell as a B-form double helix, local formation of G-quadruplex structures entails the presence of quadruplex–duplex junctions (QDJs), which inevitably are abundant in the genome. Q–D junctions may be formed during biological processes associated with the unwinding of a putative G-quadruplex forming sequence either internally, as part of a long self-complementary quadruplex loop, or externally, at the transition from quadruplex to the canonical B-type double-helical structure [17]. As represented in Fig 1, the arrangement of the duplex on the G4 core may either result in a coaxial or orthogonal orientation.

A pivotal work from Lim and Phan explored the incorporation of a duplex hairpin into the various geometries of a quadruplex core, by high resolution NMR investigations of quadruplex-duplex constructs [18]. Relevant examples of Q–D junctions have been identified as potential pharmacological targets, such as the viral HIV LTR-III sequence [19]. Notwithstanding the attractive prospects, the design of ligands binding at Q–D junctions is still an under-investigated area and few experimental studies have been reported so far.

Initial strategies employed hybrid ligands designed by simply linking a quadruplex specific moiety to a duplex minor groove binder [20,21]. However, this approach may suffer from poor development perspectives, mainly because of the molecular weight of the conjugates and their consequent low druggability.

More recent approaches assumed that Q–D junctions may by themselves constitute high-affinity binding sites for known G4 specific ligands, if compared to the “classical” stacking at exposed outer tetrads of the G4 structure.

Aminomethyl-substituted aromatic hydrocarbons, as well as indoloquinoline and pyridostatin derivatives, were able to act as Q–D junction binders, resulting in a in-depth structural characterization of their binding mode [22–26].

These pioneering studies highlighted the pivotal role of extended planar aromatic ring systems, able to cover part of the G-tetrad and bind the Q–D junction. Although the major contribution to the binding is given by vertical π–π stacking with the outer tetrad, additional horizontal electrostatic and hydrogen bond interactions with the interfacial base pairs further stabilize the complex in the case of paired bases in-plane with the ligand. Alternatively, intercalation of the ligand between the outer tetrad and a duplex base pair at a Q–D junction may occur if vertical stacking and electrostatic interactions overcome the energetic penalty associated with the unwinding at the interface, responsible for the formation of the binding pocket [27]. For these reasons, the presence of appropriate side arms on the planar aromatic core structure of G4 ligands is considered highly beneficial for the binding affinity to the Q–D junction, allowing additional stabilizing interactions (short-lived electrostatic, hydrogen bond, and/or van der Waals forces) with the grooves or the loop regions. However, poor selectivity could be due to appended side chains favorably interacting with the duplex minor groove [28]. Rather, the Q–D selectivity seems to significantly benefit from an extension of the side chain towards the exposed part of the G-tetrad at the junction. As observed by the NMR analysis of unsubstituted cryptolepine, side chains may effectively restrict ligand dynamics and exchange between different ligand alignments to fix a major ligand orientation [28].

**Fig 2:**
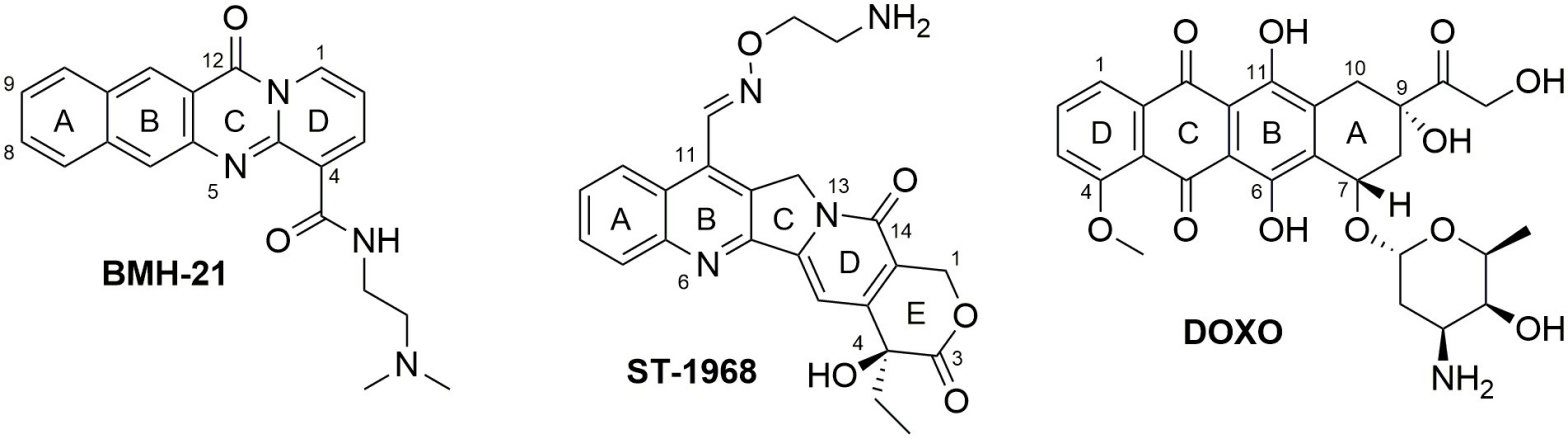
Molecular structures of BMH-21, ST-1968 and DOXO.

With the aim to identify new scaffolds able to interact with Q–D junctions, we investigated three structurally diverse polycyclic compounds, known to interact with the quadruplex moiety: BMH-21, namitecan (11-carbaldehyde *O*-(2-aminoethyl) oxime camptothecin, hereafter referred as ST-1968), and doxorubicin (DOXO) (Fig 2) [29–33]. All the three compounds bear either one short aminoalkyl moiety or an aminoglycosyl group, which should play a role in increasing the interactions at the level of the junction, as reported above.

## Materials and methods

### Oligonucleotide and ligands

The oligonucleotide LTR-III, 5’-GGGAGGCGTGGCCTGGGCGGGACTGGGG-3’, was synthesized in 1 μmol scale on an Applied Biosystems DNA/RNA 3400 synthesizer by solid-phase 2-cyanoethylphosphoroamidite chemistry. After the synthesis LTR-III was passed through a cation exchange, Dowex 50WX2 resin, to exchange the counter ion to sodium form and then desalted with a Sephadex (NAP-10) G25 column.

The compounds BMH-21 and ST-1968 were synthesized as previously described [34,35]. The corresponding hydrochlorides were prepared by treatment with 4M HCl in methanol. Doxorubicin hydrochloride was purchased from Sigma-Aldrich (CAS: 25316-40-9). The aglycone of doxorubicin (doxorubicinone) was obtained by hydrolysis with 0.2M HCl, at 90 °C for 40 min.

### Docking studies

The four ligands were prepared for docking by adding charges and hydrogens and the resulting 3D-structures were energy minimized. The structural models of LTR-III were obtained from the RCSB Protein Data Bank (PDB access code: 6H1K). All studies were conducted using the "open" conformation, *i*.*e*. the 1.3 conformation among those present in the downloaded NMR ensemble.

The interactions of all the four ligands with the LTR-III model were analyzed using YASARA (Yet Another Scientific Artificial Reality Application) Structure software [36] which employs the use of AutoDock Vina molecular docking algorithm [37]. The molecular docking experiments were done using the dock_run.mcr macro from YASARA structure with 100 docking runs, setting the number of binding modes to 10 and the exhaustiveness of search to 8, with a maximum energy difference of 3 kcal/mol. Each docking run was ranked based on the best binding free energy (kcal/mol). The resulting complexes were analyzed and chosen based on energy and compliance with experimental data. The resulting best conformations were then optimized by the steepest descent conjugate method using the OL15 modification of the Amber force field [38], as implemented in the YASARA structure software. The resulting YOB files were converted into PDB format and used to analyze the position of each ligand and contact bases through three-dimensional visualization with BIOVIA Discovery Studio (DS) visualizer [39].

### CD and fluorescence

CD-monitored melting experiments were carried out in triplicates using a Jasco J-815 spectropolarimeter equipped with a Peltier unit for temperature control. The DNA solution was transferred to a 10-mm-path-length cell and the heating rate was set to 1 °C·min^−1^. In addition to the measured ellipticity at 280 nm, spectra were measured from 220 to 320 nm at 5 °C intervals after a holding time of 2 min. To evaluate the stability of LTR-III under different conditions, sodium cacodylate (20 mM sodium cacodylate and 70 mM potassium chloride) and phosphate (20 mM potassium phosphate and 70 mM potassium chloride) buffers were employed (S1 Fig in S1 File). The melting temperatures (T_m_) were determined by fitting a sigmoidal curve to the ellipticity values measured at 284 nm. The assumption of a two-state process was previously checked by means of multivariate analysis of the whole set of spectra measured along each melting experiment.

The molecular fluorescence spectra were measured with a JASCO FP-6200 spectrofluorometer. The temperature was set to 20 °C using a water bath. Both direct (ligand being titrated with DNA) and reverse (DNA being titrated with a ligand) titrations were performed. In both cases, the fluorescence data were analyzed by means of the Equispec software [40] to determine the stoichiometry of the resulting DNA:ligand complexes and the corresponding binding constants as mean of three replicates. This procedure has been applied previously to describe DNA:ligand equilibria [41].

### NMR studies

NMR experiments were performed on 600 MHz Bruker Avance spectrometers. LTR-III (62 OD, 0.55 mL H_2_O/D_2_O 9:1 buffer solution having 70 mM KCl and 25 mM potassium phosphate, pH = 7.0) was characterized, following the previous assignments [19], through one-dimensional (1D) hydrogen nuclear (^1^H), two-dimensional (2D) total correlation spectroscopy (TOCSY) and nuclear overhauser effect spectroscopy (NOESY) experiments acquired at 298 K, 283 K and 308 K (S1 Table in S1 File). Water suppression was achieved by excitation sculpting sequence from standard Bruker library. The oligonucleotide was heated to 85 °C for 1 min and then cooled at room temperature overnight.

For interaction studies with the ligands, a stock solution of drugs was prepared in DMSO-*d*_*6*_ at a concentration ranging from 35 to 43 mM. ^1^H NMR titrations were performed by adding increasing amounts of the drug to the oligonucleotide solution until R = [ligand]/[DNA] = 2.0–3.0 was reached. The concentration of the DMSO in the sample solution was below 10% to avoid affecting the G-quadruplex conformation.

## Results and discussion

Quadruplex-duplex (Q−D) junctions represent attractive motifs for drug targeting. In the framework of a research activity aimed at identifying new potential ligands, we focused our efforts on investigating the structural requirements for the interaction. LTR-III DNA G4 was chosen as the model system to probe the binding of selected ligands to Q–D junctions, being the major G-quadruplex (G4) occurring in the long terminal repeat (LTR) promoter region of the human immunodeficiency virus-1 (HIV-1) [42].

**Fig 3:**
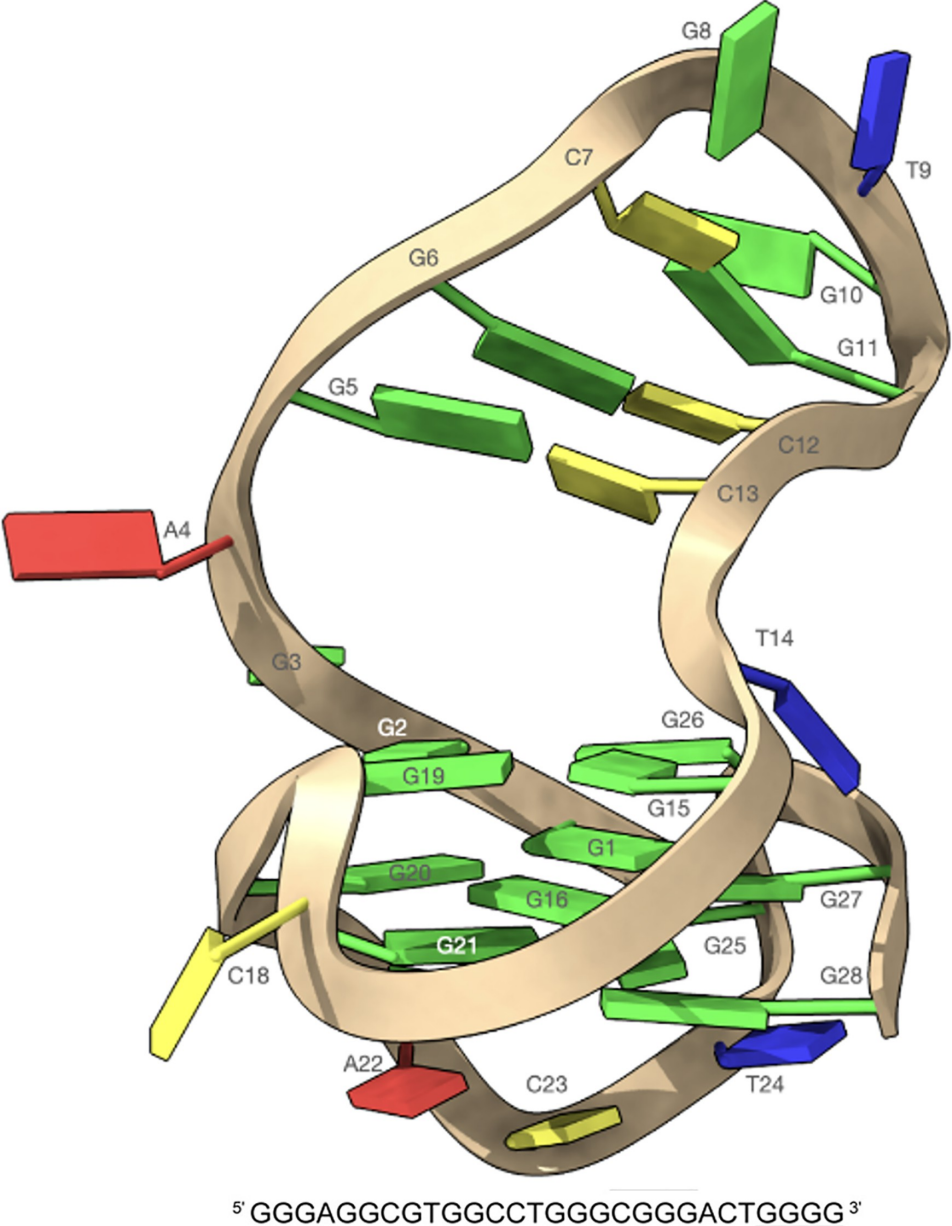
Schematic representation of a hybrid quadruplex-duplex conformation with a three-layered G-tetrad core arranged in a (3+1) topology and a long 12-nt loop forming a hairpin structure.

The structure of the LTR-III consists of a hybrid quadruplex-duplex conformation with a three-layered G-tetrad core, arranged in a (3+1) topology and a long 12-nt loop forming a hairpin structure (Fig 1, left). LTR-III is characterized by a peculiar Q–D junction which combines a highly dynamic duplex base pair at the boundary between the duplex and quadruplex moieties. Thus, the LTR-III junction structure, in the region with T14 on one strand and G3-A4 on the other of the duplex, appears to be rather floppy, providing an opportunity to be targeted by small molecules [19] (Fig 3).

The structural characteristics of LTR-III allow to define three possible binding sites for the ligands: 1) the loop duplex moiety; 2) the G-tetrad on the bottom part of G-quadruplex (G25-G28-G17-G21) and 3) the G-tetrad in the junction Q–D (G2-G26-G15-G19), including the loop region (A4, G5 and C13) in the cavity between the two units (Fig 3).

To better understand the binding abilities and the differences in the complex stabilization of the compounds with LTR-III, molecular docking methods were used [19]. The orientations of the ligands inside the binding site were further improved using the MM optimization on the resulting complexes. The docking simulations produced a set of conformers for each ligand considered, clustered according to rmsd values.

Once identified our model target, we focused our efforts on the choice of potential ligands, which were selected on the basis of previously reported literature evidence. The ideal candidates should possess an aromatic unit attached to a side chain, containing protonatable amino functionalities. Q–D junction recognition would benefit from two complementary modes of interaction: stacking of the aromatic platform onto the π-deficient surface of the G-tetrad at the interface with the duplex, plus further interactions of the ammonium moiety with the phosphate groups of the nucleotidic sequence. From an in-house library of polycyclic compounds, we initially selected compound BMH-21, whose binding to the parallel c-Myc and c-Kit quadruplex structures were structurally and thermodynamically characterized in detail by our group [32,33]. Successively, we chose for our investigation the substituted camptothecin ST-1968 and doxorubicin (DOXO), as both camptothecins and anthracyclines have been recently found to stabilize G-quadruplex structures, as well [29,31]. All the selected compounds are endowed with flat aromatic heterocyclic ring systems, prone to π-π stacking interactions. Hence, they can be considered typical G4 binding ligands, with a preference to stack on exposed outer G-tetrads, possessing however the structural requirements to act as duplex intercalators. In this framework, the side chain could represent a key motif for the interaction into the binding pocket, favoring the Q–D junction recognition.

Interestingly, both CPTs and doxorubicin have been shown to inhibit the replication of the HIV virus by an unknown mechanism of action [43–46].

### BMH-21

BMH-21 (Fig 2) is an RNA polymerase I inhibitor described as a selective binder of GC-rich sequences of DNA [47]. Recently, we have explored the ability of BMH-21 to bind the human telomeric, c-Myc promoter and c-Kit G-quadruplexes [33].

**Fig 4:**
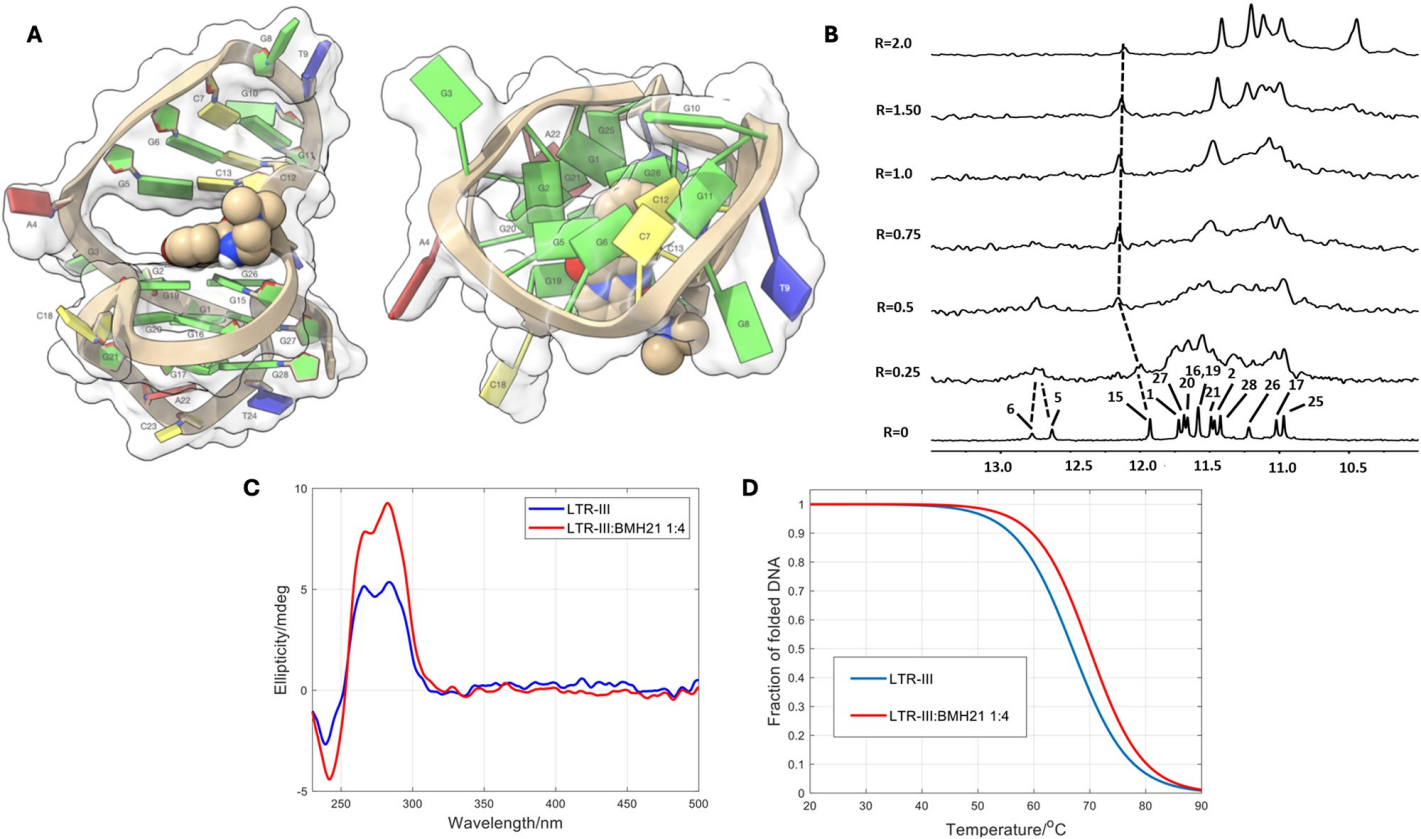
Interaction of BMH-21 with the LTR-III structure. A: The BMH-21/LTR-III complex was obtained by molecular docking and optimized by YASARA Structure [36], and it is represented as side (left) and top (right) views of the ghostly-white solvent-accessible surface (SAS) of the LTR-III target. The ligand was represented as van der Waals (vdW) spheres. The nucleotides are rendered in slabs and filled sugars (left) and mufflers and sugar as tubes (right): Cytosine in yellow, guanine in green, adenine in red and thymine in blue. Drawing was created by using the Chimera-X software [48]. B: Imino protons region of the 1D NMR titration spectra of LTR-III with BMH-21, recorded at 25 °C and different R = [BMH-21]/[DNA] ratios. C: CD spectra of LTR-III and of the 1:4 mixture with BMH-21 at 20 °C. D: Fitted fraction of folded DNA calculated from the measured ellipticity traces at 284 nm considering a two-state transition.

The docking procedure described in paragraph 2.2 was applied to define the most likely position of BMH-21 into the Q–D junction. The ligand stacks between G15 and G26 residues. In detail, the rings A-B and C-D show π-π interactions with G26 and G15, respectively. Furthermore, the complex is stabilized by a salt-bridge between G26-O1P and the quaternary nitrogen. In addition, an intramolecular hydrogen bond is formed between the amide group on the ring D and the nitrogen of ring C. The side chain with the *N*,*N*-dimethylamino group, consistently with the higher flexibility of this portion, points towards the loop A4, G5 and C13 (Fig 4A).

NMR, CD and fluorescence studies supported the presence of the hypothesized interactions at the level of the junction. The imino protons’ region (*δ* = 10–13.5 ppm) of LTR-III NMR spectrum displays several well resolved narrow signals in agreement with the literature [19]. These signals indicate the presence of three tetrads forming the well-defined G-quadruplex structure (10.5–12.5 ppm) as well as the formation of Watson-Crick hydrogen bonds (12.5–13.5 ppm) indicates the presence of a characteristic hairpin loop [19]. The chemical shift variations and line broadening of the imino proton signals were studied upon the addition of BMH-21 to the oligonucleotide solution. NMR titration experiment caused, even at a low R ratio (0.25–0.5), a significant broadening of the ^1^H imino proton signals. This was an indication that the ligand binding process is in intermediate regime and that, probably, various binding events occurred. For R ratio [BMH-21]/[LTR-III] ≥ 1.0 the signals became quite sharp (Fig 4B). The NH signals of G5, G6 and particularly G15, situated within or near the Q–D junction, appeared as the most affected in terms of chemical shift variation upon formation of the BMH-21/LTR-III complex. This behavior is in agreement with that previously reported for benzylamine derivatives [42].

The interaction of BMH-21 with the LTR-III structure was also studied by circular dichroism (CD) spectroscopy (Fig 4C and S3 Fig in S1 File). The intensity of the CD spectrum of the DNA increased slightly in a 1:4 DNA:ligand ratio, and no induced CD band was observed. These facts pointed out the preservation of the overall 3D structure upon binding. Additional information about the strength of the binding was obtained from CD-monitored melting experiments of the LTR-III structure in absence and in presence of the ligand. In general, preferential binding to the folded structure over the unfolded chain is expected to increase the thermal stability of the former (T_m_ = 66.9 ± 0.8 °C). For the DNA:ligand mixture (1:4 ratio) the determined T_m_ value was 70.0 ± 0.6 °C, i.e., the ΔT_m_ was equal to 3.1 ± 1.4 °C, in agreement with a weak stabilization of the folded DNA upon binding (Fig 4D).

The interaction of BMH-21 with LTR-III was also studied by means of fluorescence-monitored titrations. The titrations were carried out both in direct and reverse directions, i.e., the ligand being titrated with the DNA, or the DNA being titrated with the ligand, respectively. The complete multivariate data sets were analyzed using a previously described method [40,41], providing the distribution diagrams and pure spectra of all the species considered in the proposed model. For the interaction of BMH-21 with LTR-III, the analysis of the fluorescence data recorded along the direct and reverse titrations showed that the best fit was obtained when considering a 1:2 DNA:BMH-21 complex, with an overall binding constant equal to 10^12.6±0.5^ M^−2^ (S4A and S4D Fig in S1 File), which denotes a relatively weak binding, in agreement with the stabilization observed in melting experiments. As it was not possible to detect the formation of a 1:1 complex, the corresponding binding constant could not be determined. The calculated overall binding constant for the 1:2 complex pointed out to binding constants for each site around 10^6.3^ M^-1^, which denotes a relatively weak binding, in agreement with the stabilization observed in melting experiments.

### Namitecan (ST-1968)

**Fig 5:**
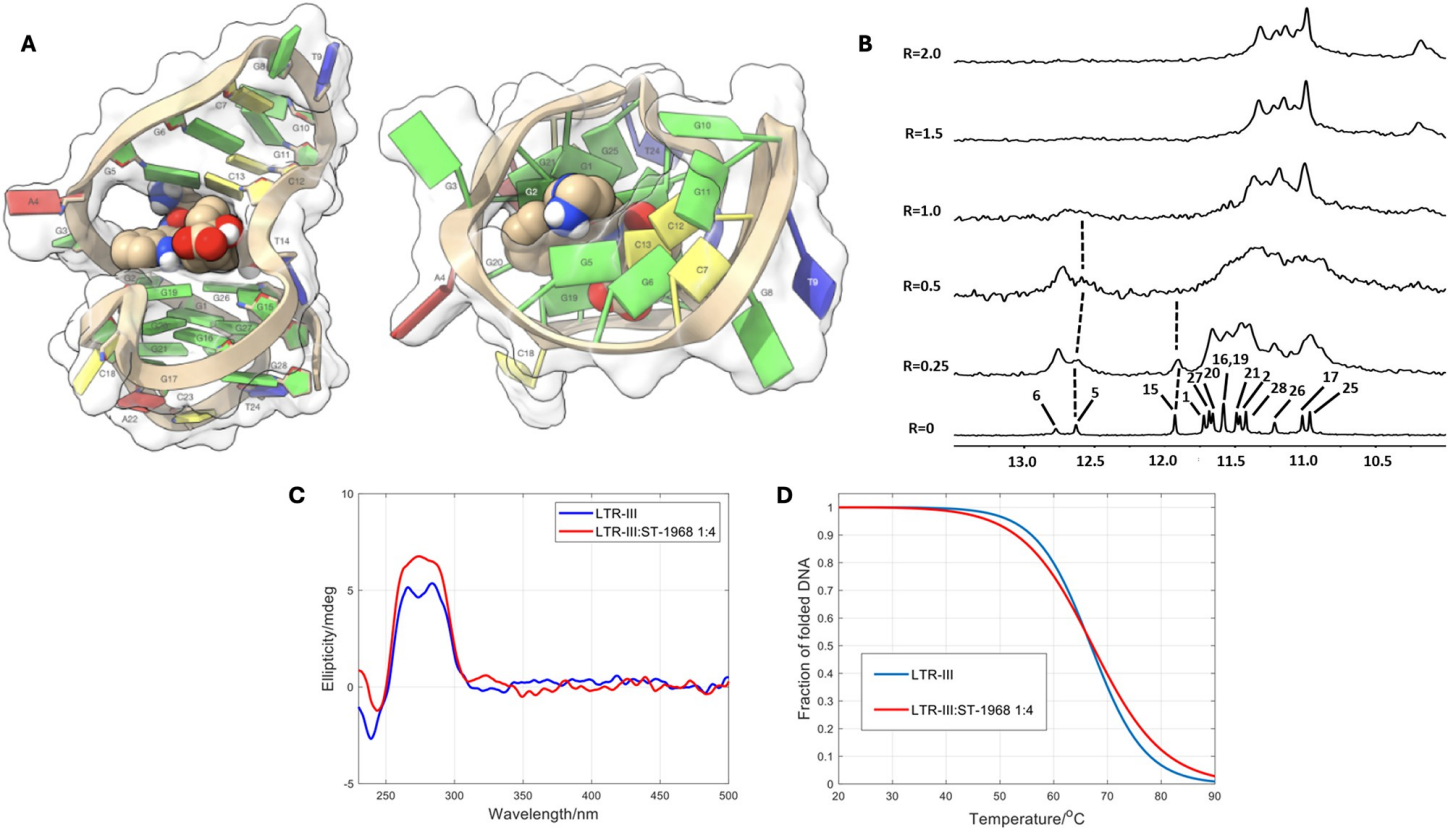
Interaction of ST-1968 with the LTR-III structure. A: The ST-1968/LTR-III complex was obtained by molecular docking and optimized by YASARA Structure [36], and it is represented as side (left) and top (right) views of the ghostly-white solvent-accessible surface (SAS) of the LTR-III target. The ligand was represented as van der Waals (vdW) spheres. The nucleotides are rendered in slabs and filled sugars (left) and mufflers and sugar as tubes (right): Cytosine in yellow, guanine in green, adenine in red and thymine in blue. Drawing was created by using the Chimera-X software [48]. B: Imino protons region of the 1D NMR titration spectra of LTR-III with ST-1968, recorded at 25 °C and different R = [ST-1968]/[DNA] ratios. C: CD spectra of LTR-III and of the 1:4 mixtures with ST-1968 at 20 °C. D: Fitted fraction of folded DNA calculated from the measured ellipticity traces at 284 nm considering a two-state transition.

The considered CPT derivative ST-1968 (Fig 2) possesses a planar fused-ring system consisting of 6/6/5/6/6 membered ring system, including a lactone moiety which is prone to hydrolysis under physiological conditions [49]. Dey and Warner experimentally determined that the lactone (neutral) and carboxylic (as a negative ion) forms of CPT are present in the molar ratios of 54:46 and 12:88 at pH 6.50 and 7.40, respectively [50]. Based on these findings, both forms of ST-1968 –lactone and carboxylate–were used in the molecular modeling studies. However, only the carboxylate form of ST-1968 data is discussed, since at the used pH conditions the presence of ST-1968 lactone form is minimal (data are reported in S2 Fig in S1 File). In the complex with LTR-III, ST-1968 is positioned with ring D near the center of the tetrad G2-G26-G15-G19, while the rings A and B stack G2 by π-π interactions. Moreover, the quaternary nitrogen of ST-1968 forms two hydrogen bonds with G5N7 (2.05 Å) and G5O6 (2.51 Å). In this conformation the hydroxyl group on ring E, derived from the hydrolysis of the lactone moiety, forms a hydrogen bond with H1 G15 (2.42 Å) and an intramolecular hydrogen bond with the OH in position 4 (2.40 Å). The pattern of interactions is completed by two weak hydrophobic bonds (π-alkyl) between the ethyl group in position 4 and G5 and C13 units. The carboxylic group points away from the loop C13-G15 in the area exposed to the solvent. The best-scoring pose of the optimized complex is shown in Fig 5A.

As displayed in Fig 5B, even at a low R ratio (0.25–0.5), all the imino proton signals in the ^1^H-NMR spectra showed a significant line broadening upon titration with ST-1968. Also in this case the behavior suggested that multiple species were present at intermediate exchange regimes between the free and the bound states. With further addition of ST-1968 (R = [ST-1968]/[LTR-III] ≥ 1.0) to the LTR-III solution, the signals belonging to the imino protons of the tetrads became sharper (Fig 5B). As in the case of BMH-21, despite the generalized broadening, the NH signals of G5, G6 and G15 units, situated in or near the Q–D junction, appeared more affected in intensity and/or in the chemical shift variation. These data agree with a concomitant binding of ST-1968 to the Q–D interface and into the duplex stem.

The interaction of ST-1968 with the LTR-III G4 was further investigated by CD spectroscopy, highlighting that LTR-III spectrum hardly changed in the presence of the ligand in a 1:4 DNA:ligand ratio, whereas no induced CD band was observed (Fig 5C). These outcomes point out to a small modification of the LTR-III structure upon binding, without any dramatic conformational changes. The analysis of the set of spectra measured along the melting of LTR-III showed that the unfolding of the hybrid structure took place in just one step (S3 Fig in S1 File). Accordingly, the change of the measured ellipticity at 284 nm was fitted to a two-states transition. For the LTR-III:ST-1968 mixture (1:4 ratio), the determined T_m_ value was 67.2 ± 0.7 °C, i.e., the ΔT_m_ was equal to 0.3 ± 1.5 °C (Fig 5d). This would indicate a similar binding to the folded form compared with the unfolded one [51].

For the fluorescence-monitored titrations of ST-1968 with LTR-III, the data were well-fitted to a model including a 1:2 stoichiometry (S4B and S4E Fig in S1 File). The calculated overall binding constant for this complex was 10^13.4±0.4^ M^−2^. In this case, a similar reasoning can be applied as described above for the binding constant of the 1:2 LTR-III:BMH21 complex. Taken together, all the obtained data confirm some degree of stabilization of ST-1968 at the level of the junction.

### DOXO

DOXO structurally consists of the tetracyclic quinoid aglycone adriamycinone linked to a conformationally free portion bearing a basic nitrogen, the aminosugar daunosamine.

**Fig 6:**
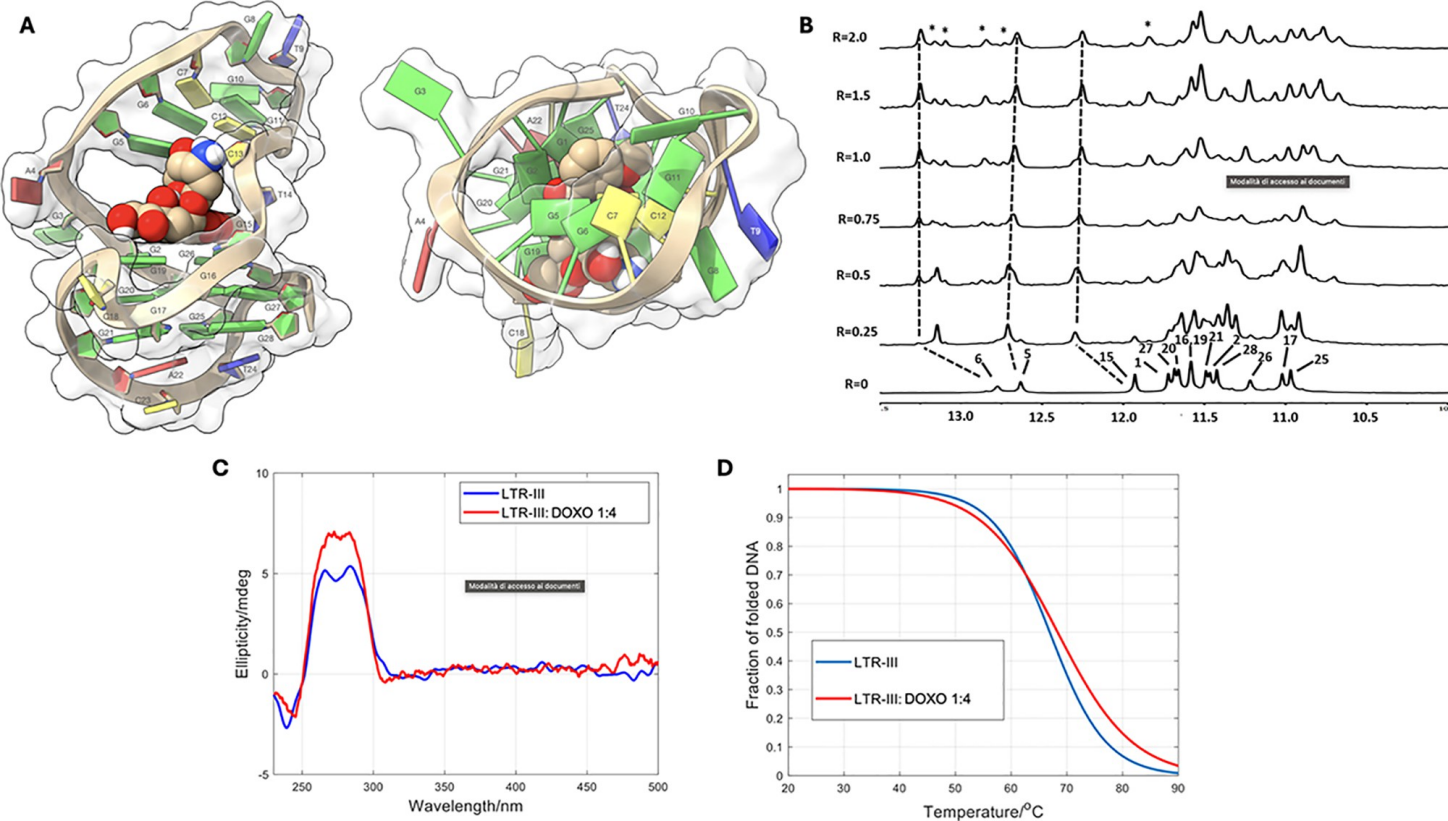
Interaction of DOXO with the LTR-III structure. A: The DOXO/LTR-III complex was obtained by molecular docking and optimized by YASARA Structure [36], and it is represented as side (a, left) and top (a, right) views of the ghostly-white solvent-accessible surface (SAS) of the LTR-III target. The ligand was represented as van der Waals (vdW) spheres. The nucleotides are rendered in slabs and filled sugars left) and mufflers and sugar as tubes (right): Cytosine in yellow, guanine in green, adenine in red and thymine in blue. Drawing was created by using the Chimera-X software [48]. B: Imino protons region of the 1D NMR titration spectra of LTR-III with DOXO, recorded at 25 °C and different R = [DOXO]/[DNA] ratios. C: CD spectra of LTR-III and of the 1:4 mixtures with DOXO at 20 °C. D: Fitted fraction of folded DNA calculated from the measured ellipticity traces at 284 nm considering a two-state transition.

The docking simulation of DOXO with LTR-III generated a complex in which B and C rings lie at the center of the G-tetrad, while the overall anthraquinone is positioned longitudinally between G26-G15 and G2-G19 in the Q–D junction, thus forming a network of π-π interactions. Moreover, the hydroxyl group in position 6 forms a hydrogen bond with G26O6 (2.67 Å). The CH_2_OH and CO in position 9 undergo hydrogen bonding with the oxygen (O4’) of the deoxyribose of G19 (2.68 Å) and with the amino group of the same unit (2.97 Å), respectively. Both the hydroxyl groups on ring C allow the formation of intramolecular hydrogen bonds with the CO of quinone moiety. The 9-OH_ax_ forms an intramolecular hydrogen bond with the O7 contributing to the orientation of the aminosugar. The NH_3_^+^ in 3’ interacts with the loop forming a salt-bridge with T14O1P, which is able to stabilize its conformation, and a hydrogen bond with C13O3’ (1.83 Å) (Fig 6A).

**Fig 7:**
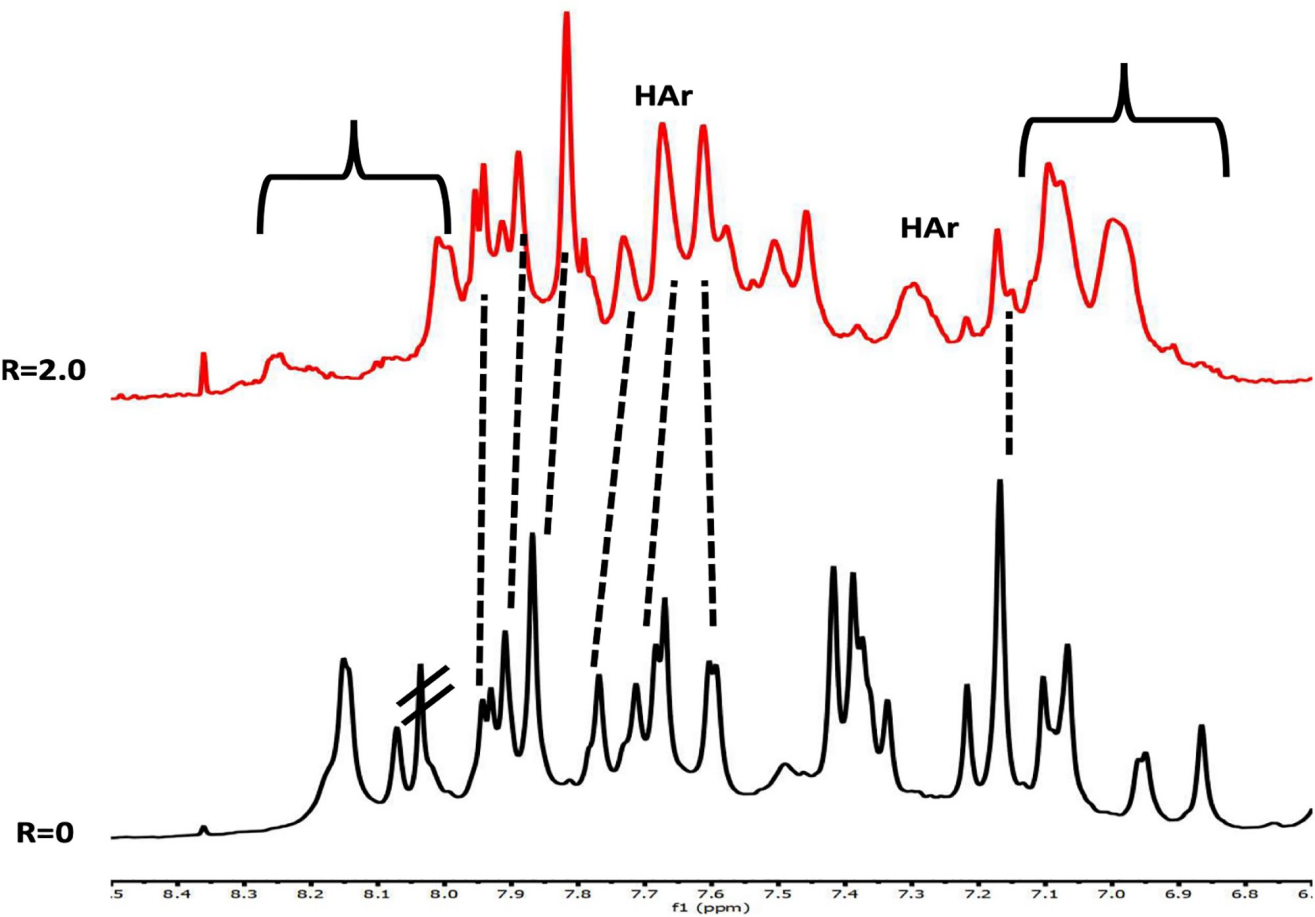
Aromatic protons region of the 1D NMR titration spectra of LTR-III with DOXO. Spectra were recorded at 25 °C; R = [DOXO]/[DNA] = 2.0.

Titration of DOXO to LTR-III resulted in the appearance, even at low ratio, of a new set of signals attributed to the complex DOXO/LTR-III in slow chemical exchange with free LTR-III. After the addition of 1 eq of DOXO, the NMR spectrum did not change, and one predominant single species was observed, together with other minor species in slow chemical exchange (Fig 6B). Following the NH resonances during the titration experiment, a down-field chemical shift change was observed for the Watson-Crick imino protons G6 and for the interfacial G15 imino upon addition of DOXO (Δδ NHG6 = +0.41, Δδ = +0.27). The down-field shift for NH imino protons of the double helix suggested that the DOXO did not intercalate between the base pairs. Indeed, our previous findings demonstrated that intercalation causes a relevant up-field shifts of NH protons [52,53]. The other NH imino protons of the G-tetrads underwent up-field shift variations (S2 Table and S5 Fig in S1 File). Upon titration with DOXO, significant changes in the 1D ^1^H-NMR spectrum were observed, in particular in the region corresponding to pyrimidine H6 and purine H8 protons of LTR-III (Fig 7). The most affected aromatic protons belonged to G21, G25 and A22, G16 and G20 located in the 3’ terminal end of the LTR-III quadruplex and in the middle tetrad, respectively. Aromatic protons of duplex hairpin (G6, C12 and C13) and aromatic protons belonging to tetrad in the junction (G26, G19 and G1), together with some aromatic protons belonging to the residues located in the loops, were affected as well. On the other hand, minor changes were detected for the remaining residues.

As described for the other two ligands, the overall structure of the DNA is preserved after interaction with DOXO, as deduced from the CD spectra (Fig 6C). For the mixture of DNA and DOXO (1:4 ratio), the determined T_m_ value was 72.8 ± 0.8 °C, i.e., the ΔT_m_ was equal to 5.9 ± 1.6 °C, which could be related to a certain stabilization of the folded DNA upon binding (Fig 6D and S3 Fig in S1 File). As in the previous cases, the analysis of the data recorded along the fluorescence titrations fit well to a model consisting of a 1:2 DNA:DOXO complex (S4C and S4F Fig in S1 File). The calculated overall binding constant for this 1:2 complex was 10^13.3±0.2^ M^−2^. Also, the 1:1 complex could not be detected nor quantified. From the calculated binding constant of the 1:2 complex, it could be deduced the existence of two binding sites with binding constants around 10^6.6^ M^-1^,in agreement with the thermal stabilization observed in presence of the ligand.

To summarize, the computational modeling studies suggested that BMH-21 and ST-1968 establish a network of interactions with the Q–D junction on LTR-III. Both complexes are stabilized by the formation of different types of intermolecular non-covalent interactions such as electrostatic, π–π stacking and hydrogen bonding with the aromatic rings and the G-tetrad in the junction. The side chains of the two compounds, having great conformational freedom, point near the loop (Figs 4a and 5a). However, due to the peculiar crescent shape of the scaffold, ST-1968 can perform interactions at the level of the loop. As for BMH-21, the association of DOXO with LTR-III is mainly driven by two major forces: electrostatic (including hydrogen and ionic bonds) and van der Waals forces (π-stacking interactions) (Table 1).

**Table 1:** Key interactions observed in the LTR-III/ligand complexes as obtained by molecular modeling. The table shows the nucleotides involved in the interactions together with the type of interactions (HB = Hydrogen Bond).

Ligand	LTR-III						
	G-tetrad in the D-Q junction				Loop region		
	G2	G26	G15	G19	T14	G5	C13
**BMH-21**		π-π	π-π, Attractive charge interaction	π-π	Attractive charge interaction		
**ST-1968**	π-π					HB	
**DOXO**	π-π	π-π, HB	π-π	π-π, HB	Attractive charge interaction	HB	HB

From our studies it emerged that the amino sugar moiety plays a role in enhancing the binding affinity by the formation of extra non-covalent interactions with the DNA bases adjacent to the binding site.

To confirm the contribution of the amino sugar to the interaction with the Q-D junction, the glycosidic portion of DOXO was hydrolyzed, and NMR titration experiments were carried out (S6 Fig in S1 File). The titration was performed by adding the aglycone to the LTR-III solution till ratio R = [aglycone]/[DNA] = 3.0. Notwithstanding the lower solubility of the aglycone in comparison with DOXO, a precipitate was observed only at R ≥ 2.0. The NH imino protons signals, as well as the aromatic proton signals, remained very broad at all the R ratios. This behavior suggests the formation of a non-specific binding to the oligonucleotide and/or a destabilization of the complex. Overall, the results confirmed that the aminosugar moiety is crucial for the binding to G-quadruplex structure. The presence of a charged residue extending within the binding site of the G-quadruplex allows the interaction with the C13 residue, towards the external groove, contributing to increase the stability of the complex.

## Conclusions

Q–D junctions may be considered novel DNA targets for selective drug targeting, due to the presence of duplex and quadruplex structures and their frequent occurrence in genomic sequences [17]. We used spectroscopic experimental approaches, combined with molecular modeling calculations, to investigate the interactions of BMH-21, ST-1968 and DOXO with the Q–D junction LTR-III. The results showed that all the selected compounds had a moderate but clear effect on the stabilization of LTR-III.

The binding of BMH-21 and ST-1968 to the LTR-III target was not associated to a significant stabilizing effect, since the ligand approach to the interface was possible only after suitable opening of the corresponding residues. From the energetic point of view, the loss of hydrogen bonds at the interface was compensated by the ligand-DNA π-π interactions. Therefore, ligand binding to the Q–D junctions of LTR-III resulted in a global process with no dramatic effects on the thermal stability. DOXO was found to be the best Q–D junction binder, as evidenced by the complex denaturation curve, which showed higher melting temperature values. In addition, it was found that DOXO binding mode was quite different from the binding mode of the other two tested compounds. Molecular modeling studies suggested that BMH-21 and ST-1968 established similar networks of interactions with the target, maintaining approximately the same position. Conversely, the DOXO aromatic moiety was placed in a significantly different manner, the anthraquinone being situated orthogonally to the axis of the double helix. (Figs 4a, 5a and 6a). The presence of the aminoglycosyl group has an important impact in the ligand binding to Q–D junctions, as observed by molecular docking experiments. This was confirmed by the NMR analysis of the aglycone binding experiments (S6 Fig in S1 File), which indicated an interaction of the aminoglycosyl group with exposed regions of interfacial tetrad of the Q–D junction. Our chemical investigations, supported by biophysical data and molecular modeling studies, provide guidelines for the identification of new scaffolds for selective Q–D recognition. Since CPTs and doxorubicin have been found to inhibit HIV virus with unknown mechanisms of action [43–46], the results may be useful to extend the search for potential antiviral agents in the management of HIV infections.

## Supporting information

S1 File (DOCX)
