# Supplementary material for: Quadruplex-duplex junction in LTR-III: A molecular insight into the complexes with BMH-21, namitecan and doxorubicin
Source: PLoS One. 2024 Jul 24;19(7):e0306239. doi: 10.1371/journal.pone.0306239 (PMC11268700; doi:10.1371/journal.pone.0306239)
Supplement: S1 File — (DOCX) [file pone.0306239.s001.docx]

**Quadruplex-Duplex Junction in LTR-III: a molecular insight into the complexes with BMH-21, namitecan and doxorubicin**

Stefania Mazzini ^1^*, Gigliola Borgonovo ^1^, Salvatore Princiotto ^1^, Roberto Artali ^2^, Loana Musso ^1^, Anna Aviñó ^3^, Ramon Eritja ^3^, Raimundo Gargallo ^4^ and Sabrina Dallavalle ^1,5^

^1^Department of Food, Environmental and Nutritional Sciences (DEFENS), University of Milan,

20133 Milan, Italy;

^2^ Scientia Advice di Roberto Artali, 20811 Cesano Maderno (MB), Italy

^3^ Institute for Advanced Chemistry of Catalonia (IQAC), CSIC, Networking Center on Bioengineering, Biomaterials and Nanomedicine (CIBER-BBN), ISCIII, 08034 Barcelona, Spain

^4^ Department of Chemical Engineering and Analytical Chemistry, University of Barcelona,

08028 Barcelona, Spain

^5^National Institute of Fundamental Studies, Kandy 20000, Sri Lanka

*** Correspondence**: [stefania.mazzini@unimi.it](mailto:stefania.mazzini@unimi.it) (S.M.)

CD spectrum and melting experiment of LTR-III in cacodylate and phosphate buffers Pag. S2

Molecular modeling on ST-1968 (lactone form) Pag. S3

Melting experiments Pag. S4

Fluorescence data for BMH-21, ST-1968, DOXO Pag. S5

2D NOESY spectra of LTR-III with DOXO Pag. S5

1D NMR titration spectra of LTR-III with aglycone Pag. S6

Selected ^1^H chemical shifts of free LTR-III Pag. S7

Selected ^1^H chemical shifts of LTR-III/DOXO complex Pag. S8

References Pag. S8


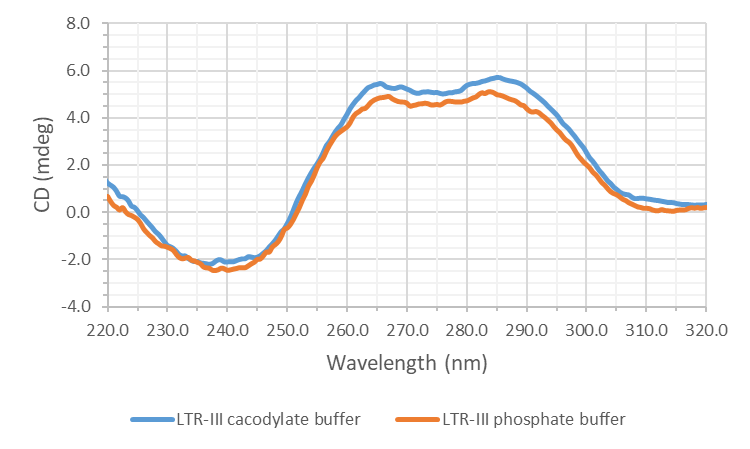

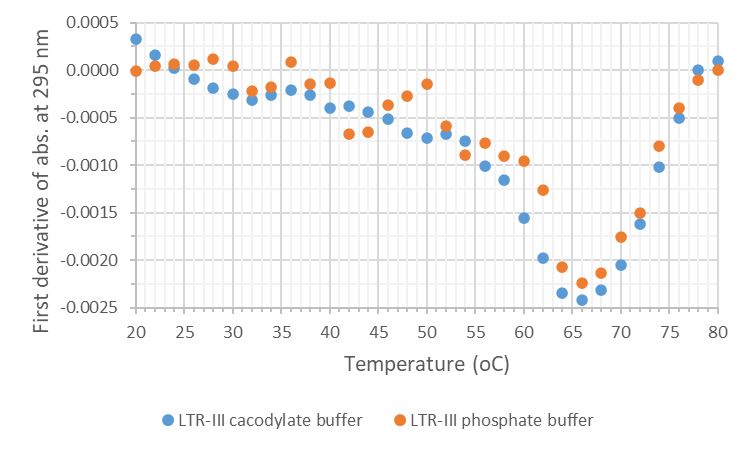


**S1 Fig**. **CD spectrum (top) and melting experiment (bottom) of LTR-III in cacodylate (blue) and phosphate (orange) buffers**.


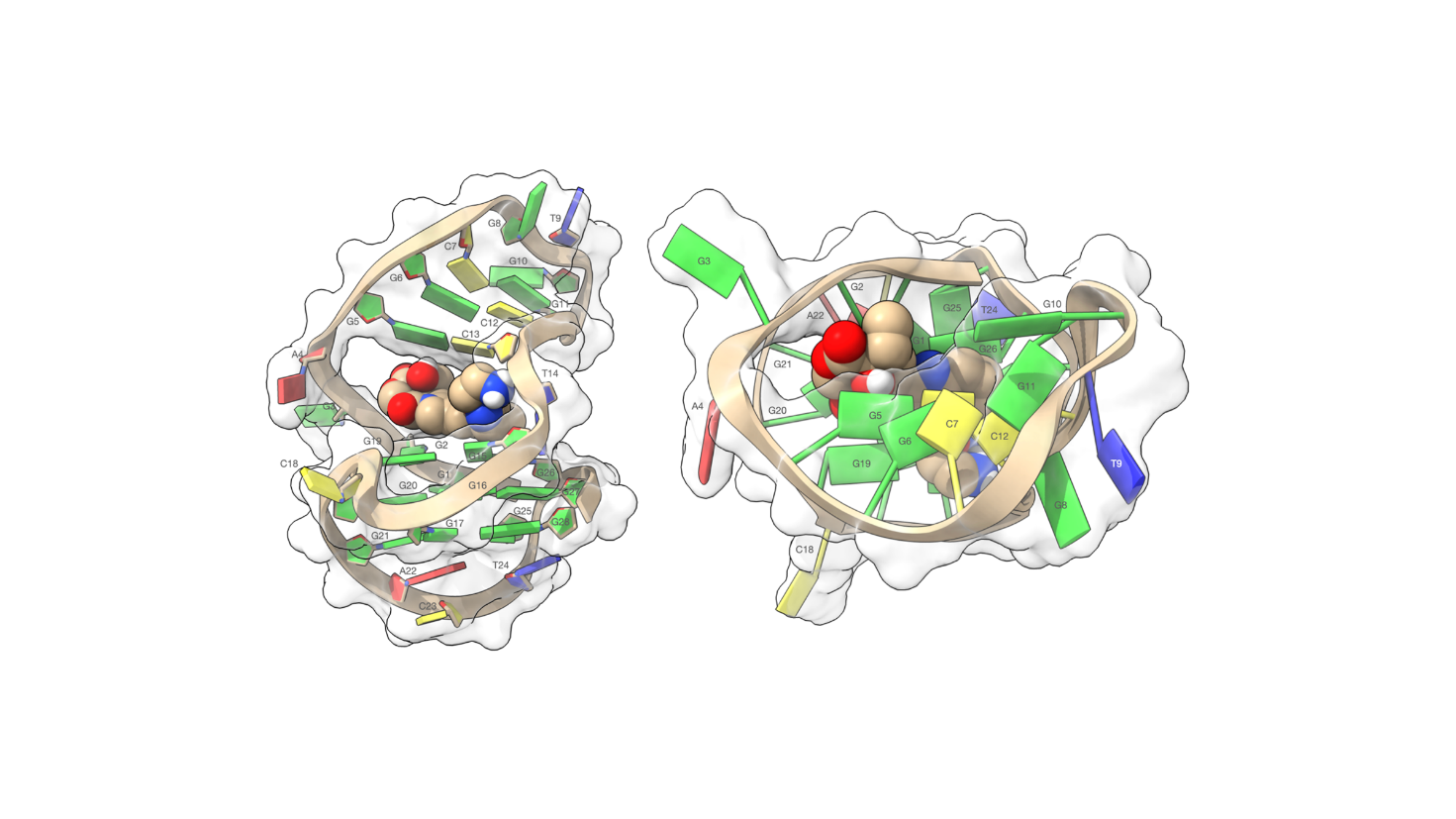
**S2 Fig**. **The ST-1968/LTR-III complex obtained by molecular docking and optimized by YASARA Structure,** [1] **considering the lactone form.** The complex is represented as side (left) and top (right) views of the ghostly-white solvent-accessible surface (SAS) of the LTR-III target. The ligand was represented as van der Waals (vdW) spheres. The nucleotides are rendered in slabs and filled sugars (left) and mufflers and sugar as tubes (right): cytosine in yellow, guanine in green, adenine in red and thymine in blue. Drawing was created by using the Chimera-X software. [2]


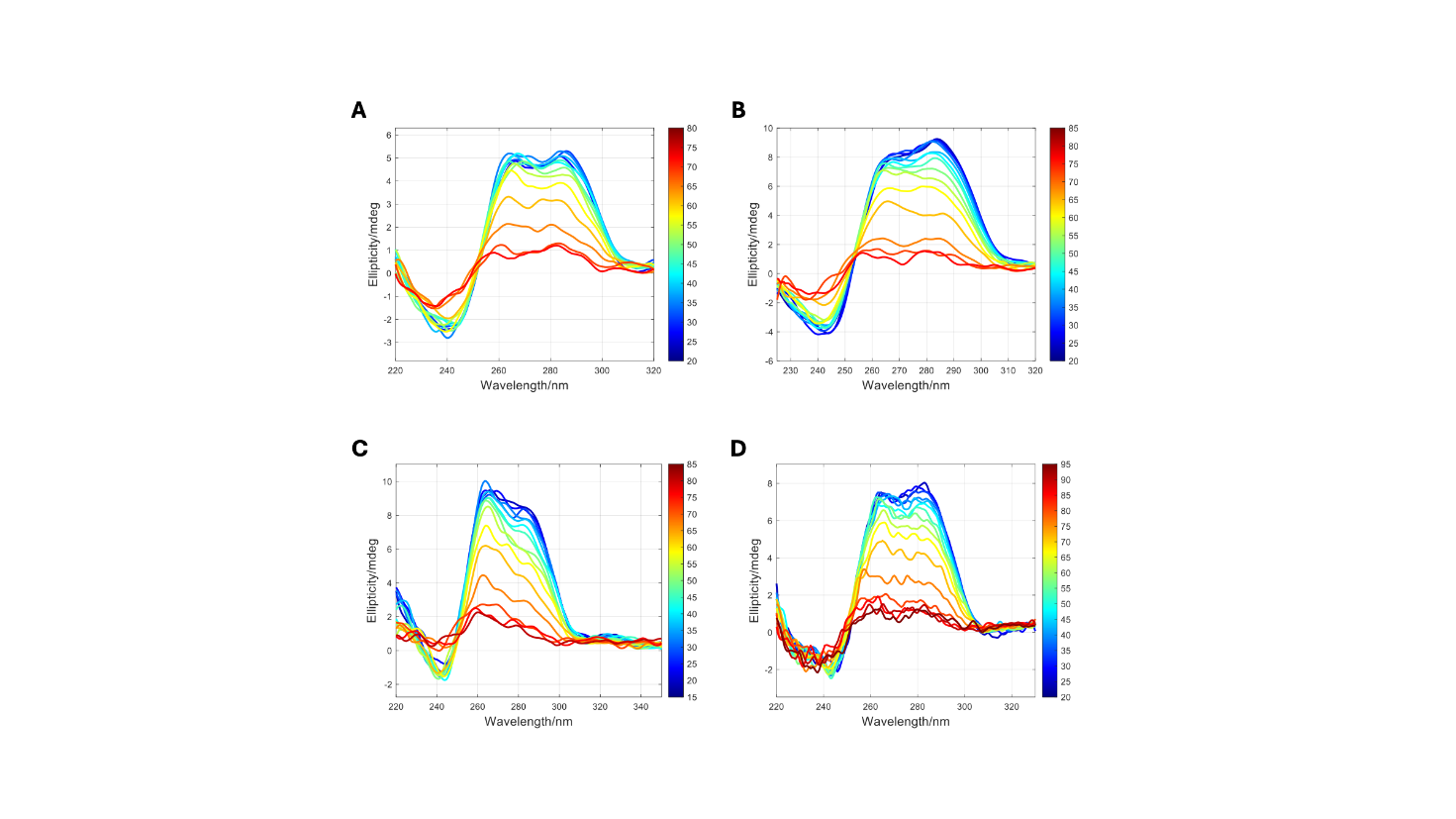


**S3 Fig**. **Analysis of the set of spectra measured along the melting of LTR-III/complexes.** Spectra measured along the melting experiments of LTR-III (**A**), LTR-III:BMH-21 1:4 (**B**), LTR-III:ST-1968 1:4 (**C**), and LTR-III:DOXO 1:4 (**D**). DNA and ligand concentration were 1.5 and 6.0 μM, respectively. Buffer conditions were 20 mM potassium phosphate and 70 mM potassium chloride.

Application of multivariate analysis (not shown) showed that these transitions could be explained by a one-step process (i.e., a two-states transition).

Values of the determined melting temperatures (T_m_) for the folding process of LTR-III calculated from the fitting of the measured ellipticity traces at 284 nm considering a one-step process:

| System | T_m_ (°C) |
| --- | --- |
| LTR-III | 66.9 ± 0.8 |
| LTR-III:BMH-21 1:4 | 70.0 ± 0.6 |
| LTR-III:ST-1968 1:4 | 67.2 ± 0.7 |
| LTR-III:DOXO 1:4 | 72.8 ± 0.8 |


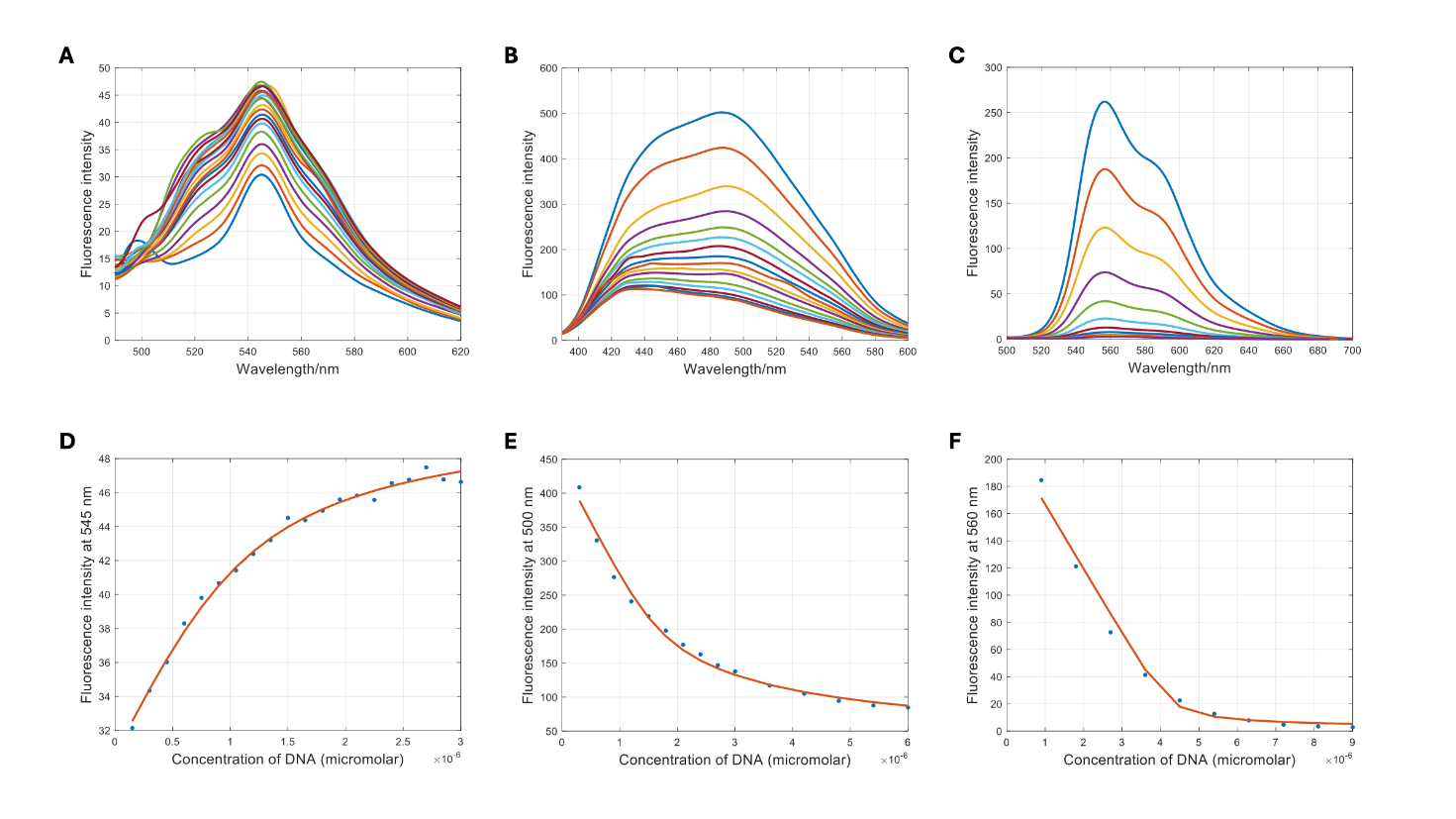


**S4 Fig**. **Analysis of fluorescence data.** Results obtained from the analysis of fluorescence data recorded along the direct and reverse titrations of BMH-21 (A, D), ST-1968 (B, E), DOXO (C, F) involving LTR-III and ligands.


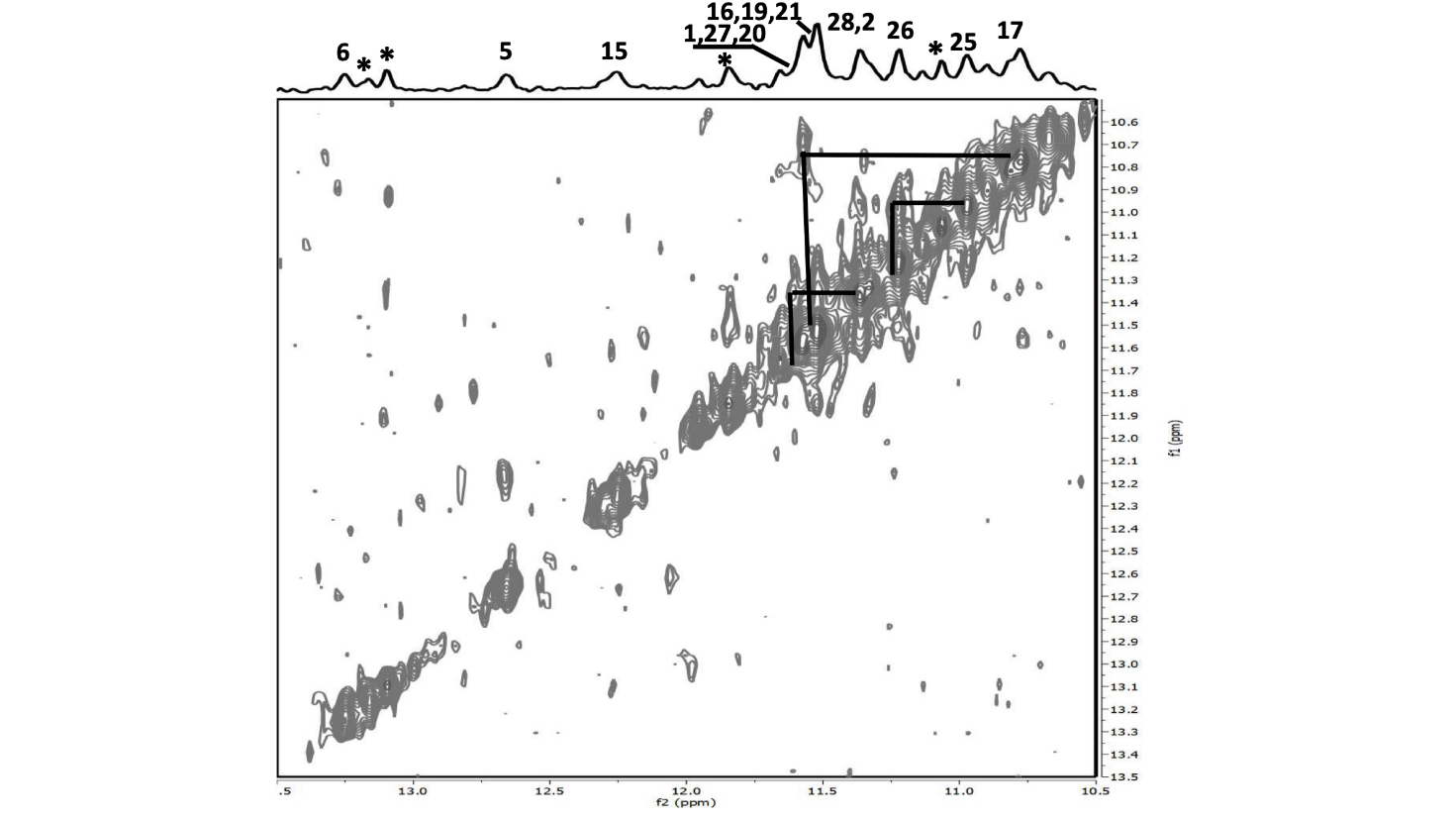


**S5 Fig. Imino-imino regions of 2D NOESY spectra (t_mix_ = 350 ms) of LTR-III with DOXO**. Spectrum was acquired at 25 °C in 90% H_2_O and 10% D_2_O, 70 mM KCl, and 25 mM K-phosphate buffer, pH 7.0, R = 2.0


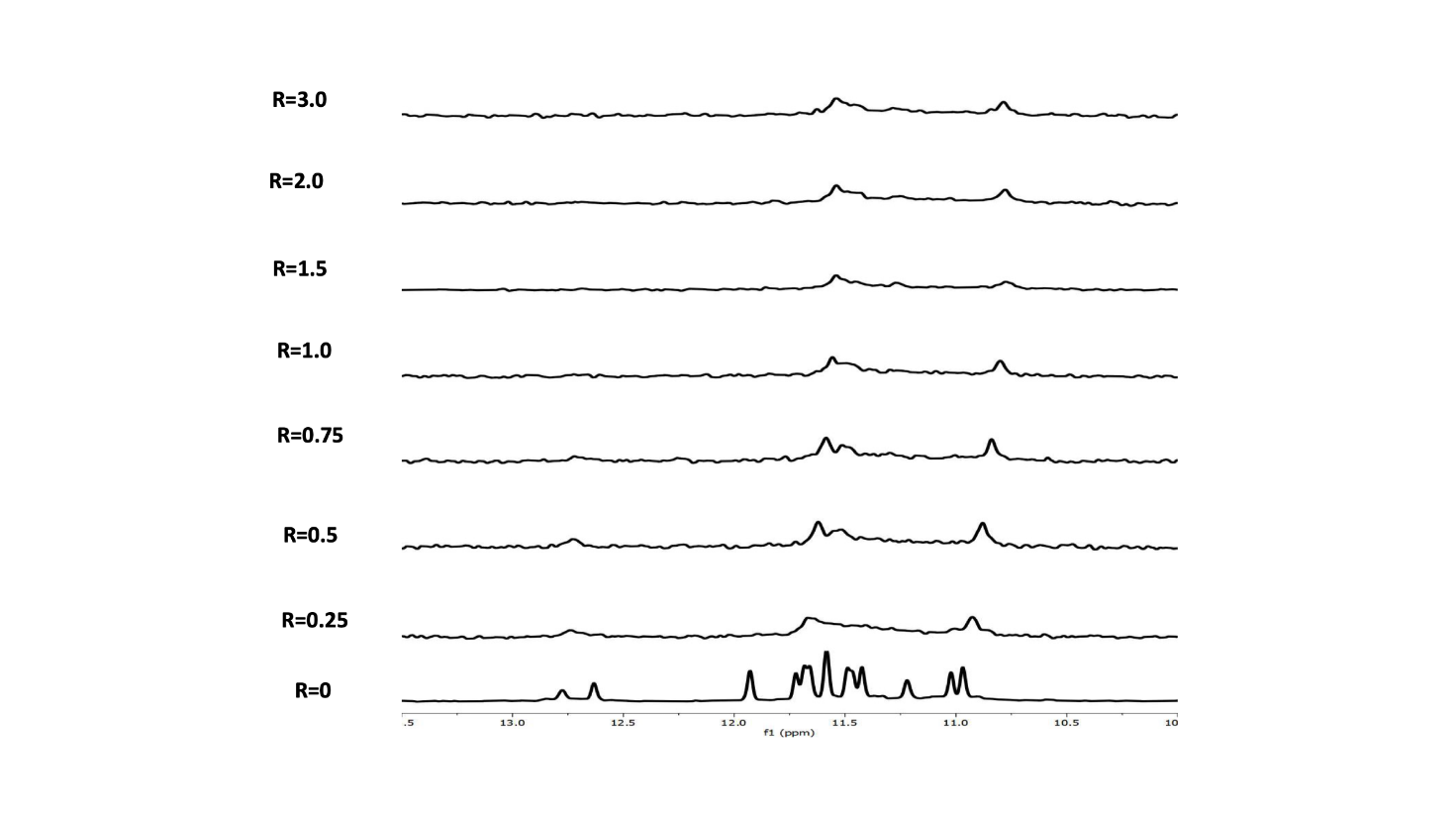


**S6 Fig**. **Imino protons region of the 1D NMR titration spectra of LTR-III with the aglycone recorded at 25 °C and different R = (ligand)/(DNA) ratios.**

**S1 Table**. **List of selected ^1^H chemical shifts (in ppm) of free LTR-III.**

| **residue** | **imino** | **H6/H8** | **H2/H5/Me** | **H1’** |
| --- | --- | --- | --- | --- |
| G1 | 11.81 | 7.23 | - | 5.86 |
| G2 | 11.55 | 7.79 | - | 5.77 |
| G3 | n.d. | n.d. | - | n.d. |
| A4 | - | 7.84 | n.d. | 5.47 |
| G5 | 12.72 | 7.67 | - | 5.49 |
| G6 | 12.87 | 7.49 | - | 5.76 |
| C7 | - | 7.02 | 5.19 | 5.87 |
| G8 | n.d. | 7.98 | - | 5.88 |
| T9 | - | 7.14 | 1.41 | 5.76 |
| G10 | n.d. | 7.74 | - | 5.55 |
| G11 | 12.95 | 7.98 | - | 5.86 |
| C12 | - | 7.44 | 5.48 |  |
| C13 | - | 7.45 | 5.57 |  |
| T14 | - | 7.24 | 1.59 | 6.09 |
| G15 | 12.02 | 7.49 | - | 6.07 |
| G16 | 11.67 | 8.22 | - | 6.07 |
| G17 | 11.11 | 7.76 | - | 6.52 |
| C18 | - | 8.09 | 6.41 (6.15) |  |
| G19 | 11.67 | 7.28 | - | 5.98 |
| G20 | 11.75 | 8.15 | - | 5.85 |
| G21 | 11.58 | 7.23 | - | 6.10 |
| A22 |  |  |  |  |
| C23 | - | 7.15 | 5.32 |  |
| T24 | - | 6.94 | 1.45 | 5.59 |
| G25 | 11.06 | 7.24 | - | 6.10 |
| G26 | 11.31 | 7.41 | - | 5.97 |
| G27 | 11.77 | 7.68 | - | 6.15 |
| G28 | 11.51 | 7.94 | - | 6.43 |

**S2** **Table. List of selected ^1^H chemical shifts (in ppm) of LTR-III/DOXO complex.**

| **residue** | **Imino LTR-III free** | **Imino LTR-III/DOXO** | **Δδ = (bound-free)** |
| --- | --- | --- | --- |
| G1 | 11.80 | 11.60 | -0.20 |
| G2 | 11.55 |  |  |
| G5 | 12.72 | 12.66 | -0.06 |
| G6 | 12.87 | 13.26 | +0.41 |
| G11 | 12.95 |  |  |
| G15 | 12.00 | 12.27 | +0.27 |
| G16 | 11.67 | 11.51 | -0.15 |
| G17 | 11.11 | 10.70 |  |
| G19 | 11.67 | 11.51 | -0.15 |
| G20 | 11.75 | 11.60 | -0.15 |
| G21 | 11.58 | 11.51 | -0.07 |
| G25 | 11.04 | 10.88 | -0.16 |
| G26 | 11.31 | 11.21 | -0.10 |
| G27 | 11.77 | 11.60 | -0.17 |
| G28 | 11.51 |  |  |

**References**

1. Krieger E, Vriend G. New ways to boost molecular dynamics simulations. J Comput Chem. 2015;36: 996–1007. doi:10.1002/jcc.23899

2. Goddard TD, Huang CC, Meng EC, Pettersen EF, Couch GS, Morris JH, et al. UCSF ChimeraX: Meeting modern challenges in visualization and analysis. Protein Science. 2018;27: 14–25. doi:10.1002/pro.3235
